# Statistical Issues and Analyses of in vivo and in vitro Genomic Data in order to Identify Clinically Relevant Profiles

**Published:** 2007-05-10

**Authors:** Laila M. Poisson, Debashis Ghosh

**Affiliations:** Department of Biostatistics, University of Michigan, Ann Arbor, MI 48109, U.S.A

**Keywords:** functional genomics, null hypothesis, in vitro, in vivo

## Abstract

In vitro experimentation provides a convenient controlled environment for testing biological hypotheses of functional genomics in cancer induction and progression. However, it is necessary to validate resulting gene signatures from these in vitro experiments in human tumor samples (i.e. in vivo). We discuss the several methods for integrating data from these two sources paying particular attention to formulating statistical tests and corresponding null hypotheses. We propose a classification null hypothesis that can be simply modeled via permutation testing. A classification method is proposed based upon the Tissue Similarity Index of Sandberg and Ernberg (PNAS, 2005) that uses the classification null hypothesis. This method is demonstrated using the in vitro signature of Core Serum Response developed by Chang et al. (PLoS Biology, 2004).

## Introduction

Integration of in vitro studies, i.e. experimental studies, with human “in vivo” gene expression studies is an area that is being considered more frequently in the functional genomic analysis of cancer. Hypotheses about cancer development, progression, and risk factors are difficult to test directly in a patient population. However, in experimental studies on cell cultures and model organisms, conditions can be specifically controlled to allow biological hypotheses to be tested. Integrating the results from such experiments with in vivo cancer signatures holds the potential to both infer activity of specific oncogenic pathways in vivo and to identify relevant effectors of oncogenic pathways.

To begin to understand the mechanisms by which oncogenes cause cancer, studies have used gene-expression profiling to identify downstream targets of oncogenic pathways in cell-culture systems. Conceptually, this involves manipulating a gene in an in vitro system, measuring the global profile using gene expression technology and then trying to relate the in vitro gene expression profile to an in vivo gene expression profile. Such an approach was taken by Lamb et al. (2003) to determine the direct transcriptional effects of the oncogene Cyclin D1. In vitro experiments were performed in which the Cyclin D1 was both over and under expressed, and global gene expression profiles were determined. Lamb et al. (2003) found that there was a significant correlation between the targets found in vitro and the ordered gene list in a human tumor dataset thus suggesting the role of Cyclin D1 regulation in tumorigenesis, another example of in vitro/in vivo gene expression data integration appears in the study of Huang et al. (2003). They developed distinct in vitro oncogenic signatures for three transcription factors: Myc, Ras and E2F1-3. These signatures were able to predict the Myc and Ras state in mammary tumors that developed in transgenic mice expressing either Myc or Ras, suggesting that specific oncogenic events are encoded in global gene-expression profiles.

Additionally, studies have used gene-expression profiling of cancerous growths induced in model organisms to examine tumor development or progression. Though model organism studies have the added difficulty of mapping orthologous genes between organisms, a difficulty not shared with tissue and cell cultures of human origin, there have been promising applications. For example, Sweet-Cordero et al. (2005) defined a KRAS induced lung cancer signature by comparing lung tumors generated from a spontaneous KRAS mutation mouse model to normal mouse lung tissue. They then correlated this KRAS lung cancer signature with gene expression profiles in human lung cancer studies and found that the mouse signature shared significant similarity with human lung adenocarcinoma but not with other lung cancer types. Next, Sweet-Cordero et al. (2005) looked for evidence of the KRAS signature in human tumors carrying activating KRAS mutations relative to wild-type tumors. Although no individual genes were significantly associated with the KRAS mutation status in human tumors, the mouse KRAS signature was significantly enriched among genes rank-ordered by differential expression in human tumors with a KRAS mutation.

It is expected that in vitro/in vivo experiments such as those described in the previous two paragraphs will become much more commonplace in the future. Thus, it is critical to address statistical issues and to develop methods for integrating in vivo and in vitro genomic data so that inferences regarding transcriptional regulatory pathways in cancer can be generated. In this article, we discuss the statistical issues of the integration of these two types of datasets. We review various existing approaches and discuss their statistical advantages and disadvantages. In addition, we outline an approach for quantifying the predictive ability of a gene expression profile determined from an in vitro experiment based on the tissue similarity approach of Sandberg and Ernberg (2005). We describe the application of the proposed methodology using in vitro data from a wound healing study conducted by Chang et al. (2004) and in vivo data from Glinsky et al. (2004), van’t Veer et al. (2002), and Beer et al. (2002). Finally, we conclude with some discussion.

## Background and Review

One class of methods that has been popular in the literature for in vitro/in vivo genomic data analysis is the following. First, one generates ordered lists of genes using the in vivo expression data. One then generates a differentially expressed gene list using the in vitro data and studies the overlap between the two lists. The seminal examples of this are in Mootha et al. (2003) and Lamb et al. (2003), which were then used as the basis of the Gene Set Enrichment Analysis (GSEA) method (Subramanian et al. 2005). We describe the GSEA methodology by briefly reviewing what was done in the Lamb et al. (2003) study. First, a list of differentially expressed genes was generated based on the comparison of Cyclin D1 overexpressing relative to wildtype (no Cyclin D1 manipulation) mammary epithelial cell lines. Next, each gene’s expression in vivo, from 190 human tumor samples of various origins, was correlated to that of Cyclin D1 and the genes were ranked accordingly. Then, a Kolmogorov-Smirnov (KS) statistic was used to determine if the in vitro differential expression list clustered within the correlation-ordered in vivo list. Since there was significant evidence of clustering, Lamb et al. (2003) determined that the in vitro-defined targets of Cyclin D1 were correlated with their respective levels in vivo. This suggests that the direct regulatory effects of Cyclin D1 may play an important role in tumorigenesis.

There are some desirable features of the GSEA method. First, it utilizes all the information available in the in vivo gene expression data; no thresholding is done in that dataset. Second, a Kolmogorov-Smirnov statistic is used for the analysis, which is a non-parametric method and thus provides some robustness. However, there are several disadvantages to GSEA as well. For instance, note that there is thresholding done in the in vitro gene expression dataset to select the differentially expressed gene set. A potential improvement to the GSEA method, to avoid this thresholding, would be the following. First, one determines the common genes in the in vivo and in vitro datasets. One then takes the scores of differential expression from the in vitro data, finds the corresponding correlation scores (correlation with Cyclin D1) in the in vivo data and examines a scatterplot of the two variables. If the association is linear, then one tests for association using the Pearson correlation coefficient between the two variables. If instead the association appears nonlinear, then one could use a smoothing-spline based test (Lin, 1997). Such an approach would give a direct test of association between the correlations in vivo and the differential expression measurement in vitro without requiring thresholding of any datasets and would still allow for a nonlinear relationship between the two variables.

Before going further, let us consider the null hypothesis under consideration in the GSEA method, or the variants proposed above. Specifically, in the Lamb et al. (2003) study they test:

### H_0_: There is no association between differential expression of Cyclin D1-overexpressed, relative to non-overexpressed, cell lines and correlation with Cyclin D1 in human tumors

**Figure 1.**
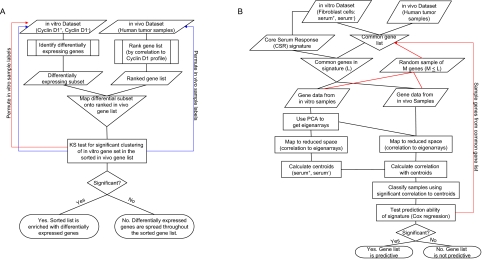
Schematic representations of the GSEA-type and TSI-type algorithms. (A) The GSEA-type algorithm is depicted along with the two suggested permutation tests (red = permutation 1, blue = permutation 2). Details of the Lamb et al. (2003) study are included for illustration. (B) The TSI-type algorithm is depicted along with the suggested permutation test (red). Details of the Chang et al. (2004) Core Serum Response signature classification are included for illustration.

The alternative hypothesis is that there is an association. In specifying the null hypothesis we uncover a more subtle disadvantage of the GSEA method—the determination of the distribution of the KS test statistic under the null hypothesis. Two variants of permutation testing have been proposed by Subramanian et al. (2005) to elucidate the distribution of the KS test statistic assuming the null hypothesis is true. In the first, the sample labels in the in vitro data are permuted, the differentially expressed gene signature is redefined, and the Kolmogorov-Smirnov statistic is recomputed based on this new signature; see Figure 1a, red. Here the implication is that the correlation between the two Cyclin D1 levels in the cell line experiment is removed by the permutation. However, this addresses the differential expression in the in vitro samples but does not address a null association with the in vivo samples. In the second version, the sample labels in the in vitro and in vivo datasets are permuted, both the in vitro differential expression signature and the in vivo correlations are redefined, and the Kolmogorov-Smirnov statistic is recomputed; see Figure 1a, blue. Again, the implication is to remove the association within the in vitro and in vivo experiments. Yet this permutation scheme still does not address the association between the in vitro differential expression and the in vivo correlation. The role of permutation testing is to simulate the distribution of the test statistic assuming that H_0_ is true; however, the two permutation schemes developed in the GSEA method do not do this. Permutation of the sample labels fails because the null hypothesis pertains to the population of genes in the two studies and not the relation of samples within a study. Additionally, Shedden (2004) suggests that permuting the sample labels of both the in vitro and in vivo data sets is not appropriate. Simply, if the permutation does not correctly model the null hypothesis correctly, then we are answering a different question than the one asked.

There is an alternative approach to the GSEA method for integrative analysis of in vitro and in vivo data, which is what we focus on in the rest of the paper. It is based on ideas of classification and clustering since the goal in many genomic studies utilizing high-throughput expression technologies is to develop a signature that can discriminate between relevant classes or groups of samples. In general, demonstration of the predictive or prognostic ability of a classification signature on independent data sets is a crucial step in the validation of that signature (Ransohoff, 2004). Thus, differential expression signatures discovered in vitro are often “validated” on independent in vivo data sets, such that the in vitro data is the training dataset and the in vivo data is the testing dataset. In this validation setting, the null hypothesis that we wish to test is the following:

### H_0_ ^class^: There exists no set of genes derived from the in vitro gene expression dataset that can predict clinical outcome in the in vivo expression data

The alternative is that at least one set of genes derived from the in vitro data is predictive. Notice that this null hypothesis is different from the null hypothesis described for the GSEA method. For clarity, we will refer to H_0_ ^class^ as the classification null hypothesis.

An advantage of the classification null hypothesis is that permutation testing becomes possible here. In particular if H_0_ ^class^ is true, then any set of genes derived from the in vitro expression profile data will have no ability to separate samples in the in vivo expression dataset with regard to a clinical outcome. Thus, we can take random sets of genes from the in vitro data and apply the classification algorithm of interest. If the classification null hypothesis is true, then all sets of genes, including the derived signature, should provide equal prediction performance.

The classification null hypothesis has motivated the following algorithm that we have used in our previous work (Varambally et al. 2005). Here we are considering the genes common to the in vitro and in vivo expression datasets.

1. Derive a gene signature from the in vitro gene expression data;
2. Select those genes from the in vivo expression data that are included in the in vitro signature and cluster the samples from the in vivo expression data into two groups using hierarchical clustering with average linkage clustering and Euclidean distance;
3. Calculate the log-rank statistic for survival between the two groups of patients;
4. Let L denote the size of the gene list in 1. Randomly choose L genes from the in vitro data as the gene signature. Continue with steps 2 and 3 above.
5. Repeat steps 2–4 1000 times. Calculate the proportion of datasets in which the log-rank statistic is greater than the one calculated initially from the signature in step 1.

The proportion calculated in step 5 will be the permutation p-value under the classification null hypothesis. This permutation scheme will form the basis of assessing significance for our proposed analytical scheme described in the next section. We note that one could also modify the GSEA procedure in a similar way, as shown in Lamb et al. (2003), such that we randomly draw the gene set from the in vitro data rather than assessing differential expression based on permuted sample labels. Unfortunately, Shedden (2004) shows that when one does not account for gene-gene correlation, the resulting test statistic can be too liberal by as much as 10 times.

Notice that a limitation of the classification null hypothesis is that the alternative hypothesis states that there exists at least one signature from the in vitro expression data that is predictive in the in vivo expression data. In fact the experimentally derived gene list need not be a unique classifier. It has been recently noted that there are likely many gene signatures that have similar predictive power (Ein-Dor et al. 2005; Fan et al. 2006). It may be due in part to genetic redundancy or to the high correlation of genes within a pathway. Yet if the in vitro gene signature is able to predict prognosis better than a randomly selected set of genes we expect that there is biological significance to that signature. Thus permutation testing helps us to determine if the gene set derived from the in vitro experiments is of interest for further study of its biological relevance.

## Proposed methodology for in vitro/in vivo analyses

The paper of Sandberg and Ernberg (2005) considers the relationship between the gene expression of in vitro cell cultures and their respective in vivo tumor samples. To that end they developed an algorithm for comparing gene expression values across experiments that they call the tissue similarity index (TSI). We use that algorithm here to compare the in vivo tumor samples to the in vitro samples of a lab experiment.

The algorithm of Sandberg and Ernberg (2005) is as follows; see Figure 1b. Principal component analysis is run on the covariance matrix of gene expression for genes in the in vitro dataset. Data are scaled across arrays so that each gene has a mean expression of zero and a unit standard deviation. The resulting eigenarrays (eigenvectors) are stored. To project the in vitro gene expression into the reduced dimensional space, created by the eigenarrays, calculate the correlation between each eigenarray and each in vitro sample array. The consensus signature for each experimental condition (serum induced and serum independent) is represented by its median centroid in the reduced space.

To integrate the in vivo data, first map the in vivo samples into the same reduced space of the in vitro samples by again calculating the correlation between each eigenarray and in vivo sample array. To maintain scale in this correlation, the tumor samples are also standardized so that each gene has a mean expression of zero and a unit standard deviation. The distance between the in vivo tumor sample and each of the two consensus signatures, i.e. centroids, is calculated using Pearson correlation. Samples are classified with the experimental condition with whose centroid they correlate best.

There are several differences between their and our implementations of TSI. First, in contrast to Sandberg and Ernberg (2005), we use positive statistical significance of the TSI to determine classification, thus allowing some samples to remain unclassified. In their paper they used an ad-hoc threshold value for TSI score, delineating moderate and high correlation groups. It is natural to believe that some of the in vivo samples will not correlate well with the in vitro conditions. These unclassified samples may actually be informative in that they define a subset of cases which do not meet our expectation as developed in the hypotheses tested in vitro. Second, the goal of the Sandberg and Ernberg (2005) paper was qualitative assessment of cell line gene expression relative to in vivo tumor gene expression, thus they do not address the issue of statistical significance of their method. However, the classification provided by the TSI can be tested for prognostic or diagnostic value depending upon the study goal.

Since the gene signature on which the classification is based is determined from the in vitro data, and does not use the in vivo data, the statistical significance of any tests on the in vivo data can be accepted without bias. This is an example of using the in vitro data for the training dataset and the in vivo data for the testing dataset. Indeed, if this in vivo validation is not at least marginally significant it is not of interest to proceed further to test the classification null hypothesis.

Using the TSI method, we develop a classification scheme from the in vitro signature. The null hypothesis of interest is again the classification null hypothesis as presented above. We thus propose the use of a permutation test to determine the utility of the gene signature in its classification ability. In the following we slightly modify the permutation test procedure described in the previous section to account for gene-gene correlation within the in vitro gene signature. Specifically, as it is likely that genes within a pathway are correlated, it is reasonable to assume that the significantly differentially expressed genes that comprise the in vitro signature are correlated. Shedden, (2004) showed that this correlation can lead to invalid p-values. In the classical genetics setting, Nyholt, (2004) shows that permutation tests that do not account for this correlation can be misleading and proposes a simple adjustment. In essence, rather than randomly selecting L genes in each cycle of the permutation test, only M (M ≤ L) genes are selected, where M is calculated to be the effective number of independent genes in the gene signature (Nyholt, 2004); see Figure 1b.

Finally, the permutation test for the TSI analysis has two interesting attributes against which the classification signature is compared. Specifically, in permuting the data, the TSI scores are recalculated using the randomly selected gene list and with each randomly selected set of genes there is a possibility of unclassified samples. Thus the classification is compared to: (1) the measure of association with predictive factors in vivo, and (2) the percentage of unclassified samples in vivo.

## Results

### Data acquisition and preparation

For the purpose of demonstration we use, as the in vitro derived signature, the wound healing signature of Chang et al. (2004). Derived from cultured fibroblasts in the presence and absence of serum components, the wound healing signature is composed of 573 genes that are differentially expressed in response to serum. We consider the wound healing signature, or Core Serum Response (CSR), as the in vitro basis of classification of in vivo tumor samples—prostate tumor samples (Glinsky et al. 2004), breast tumor samples (van’t Veer et al. 2002), and lung tumor samples (Beer et al. 2002)—into good and bad prognosis groups.

The fibroblast gene expression data (Chang et al. 2004) was downloaded from the Stanford Microarray Database (SMD, http://smd.stanford.edu/cgi-bin/publication/viewPublication.pl?pub_no=293) (platform: cDNA microarray, 50 samples). The data were normalized using loess normalization by print block within array (Yang et al. 2002). Interarray variability was accounted for by scaling using the MAD (median absolute deviation). Missing data was imputed using KNN (K-nearest neighbors) imputation as implemented in the pam.r package (Hastie et al. 1999; Troyanskaya et al. 2001).

Localized prostate tumor probe-set level expression measures and recurrence free survival information (Glinsky et al. 2004) were obtained from the Sidney Kimmel Cancer Center website (no longer posted at time of submission) (platform: Affymetrix U95Av2, 295 samples). Lung adenocarcinoma probe-set level expression measures and overall survival information (Beer et al. 2002) were obtained from http://dot.ped.med.umich.edu:2000/pub/Lung/index.html (platform: Affymetrix HUgenFL, 86 samples). Sporadic breast cancer expression data and recurrence free survival information (van’t Veer et al. 2002) were obtained from http://www.rii.com/publications/2002/vantveer.html (platform: Aglient Hu25K, 78 samples). Each of these experiments was normalized by global scaling per array. No imputation was done for missing data in the tumor sample data sets.

Unigene Cluster ID number was used to map genes between platforms. Annotation information was acquired from SOURCE (Diehn et al. 2003). If, for a given platform, multiple measurements were represented by the same Unigene Cluster ID, these expression values were averaged within array, thus allowing one-to-one mapping of genes between platforms. Genes were mapped to Unigene Cluster ID from GenBank Accession number if available (Chang et al. 2004; Glinksy et al. 2004; van’t Veer et al. 2002) or from Unigene Symbol (Beer et al. 2002)

## Application of the TSI based classifier

**Figure 2.**
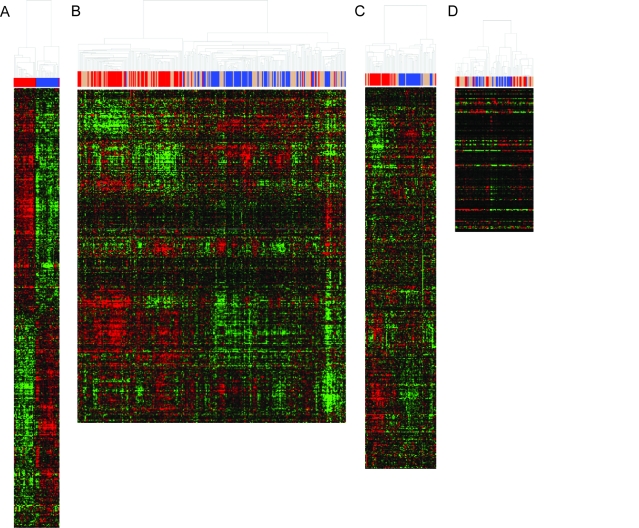
Heatmap of core serum response genes in each of the four data sets considered. Red are serum induced samples, blue are serum independent samples, and tan are unclassified samples. (A) Expression of 484 Unigene mapped core serum response genes in the 50 in vitro samples of the primary experiment (Chang et al. 2004). (B) Expression of the 376 core serum response genes in the 295 prostate tumor samples (Glinsky et al. 2004) (C) Expression of the 421 core serum response genes in the 78 van’t Veer samples (van’t Veer et al. 2002) (D) Expression of the 158 core serum response genes in the 86 Beer samples (Beer et al. 2002).

**Figure 3.**
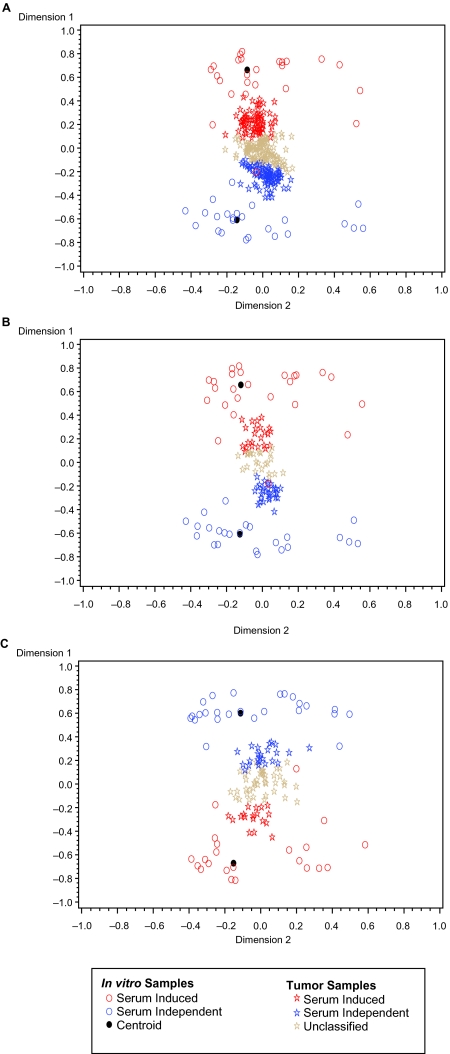
In vitro samples and tumor samples are plotted for the first two dimensions of the reduced space. (A) 55% of the prostate tumor samples are classified: 78 as serum induced, 83 as serum independent (B) 69.3% of the breast tumor samples are classified: 27 as serum induced, 27 as serum independent. (C) 48.9% of the lung tumor samples are classified: 18 as serum induced, 24 as serum independent.

The classifier was built using the CSR in vitro signature and the TSI algorithm, described in the previous section and in Figure 1b. The classifier was built for each of the three in vivo experiments using only those genes in the CSR signature that were common to both the in vivo and in vitro experiments; see Table 1 and Figure 2. All 50 eigenarrays were used for the TSI classification algorithm and classification is based on significant positive correlation with one of the two CSR group centroids. Figure 3 plots the first two dimensions of this reduced space for each of the three tumor types. The cell cultures that were grown in the presence of serum were considered to be serum induced, whereas those grown without serum components were serum independent. In vivo samples that correlate significantly (p < 0.05) with the composite serum induced signature, i.e. centroid, are classified as serum induced. Likewise, those in vivo samples correlating significantly with the centroid of the serum independent samples are labeled serum independent. In vivo samples that do not correlate significantly with either centroid remain unclassified. In Figure 3, the tumor samples are colored according their classification and the in vitro samples and centroids are included for reference.

**Figure 4.**
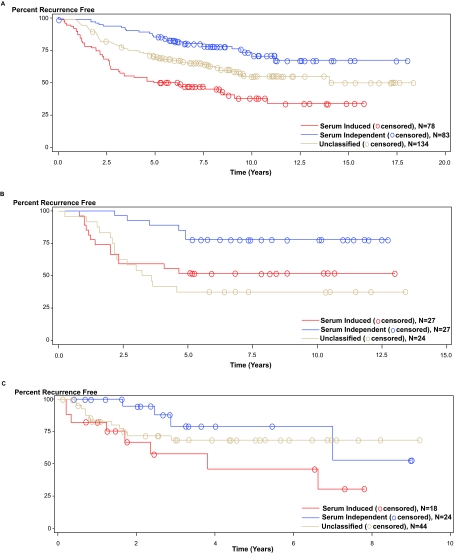
Survival of classified tumor samples demonstrating that patients with samples in the serum induced class are likely to have a worse prognosis. Both the classified and unclassified samples are included in these plots. The log-rank statistic p-values from Kaplan Meier estimation are given for the separation of the classes without and with inclusion of the unclassified samples (A) recurrence free survival in prostate cancer: p < 0.0001; p < 0.0001, (B) recurrence free survival in breast cancer: p = 0.0207; p = 0.0078, (C) overall survival in lung cancer: p = 0.0352; p = 0.0789.

According to H_0_ ^class^, we wish to see if the in vitro derived CSR signature has prognostic ability in vivo. Thus the prognostic ability of the CSR signature as a classifier was tested using univariate Cox regression; see Table 2. The TSI score was incorporated through its discrete classification of the in vivo samples, as described above. Figure 4 contains the Kaplan-Meier survival curves for this discrete classification. The red and blue curves represent the serum activated and serum independent classifications, respectively. Log-rank statistics on the Kaplan-Meier estimates indicate that there is a significant separation between the curves for the prostate tumors (p < 0.0001), the breast tumors (p = 0.0207), and the lung tumors (p = 0.0352). The tan curve shows the survival of those samples that did not significantly correlate with either the serum activated or serum independent profiles and are thus left unclassified by the TSI algorithm. When this unclassified group was included in the Log-rank test of survival curve separation the prostate cancer and breast cancer samples remained significant (p < 0.0001 and p = 0.0078, respectively) whereas the lung cancer samples were marginally significant (p = 0.0789).

### Permutation testing of H_0_ ^class^

**Figure 5.**
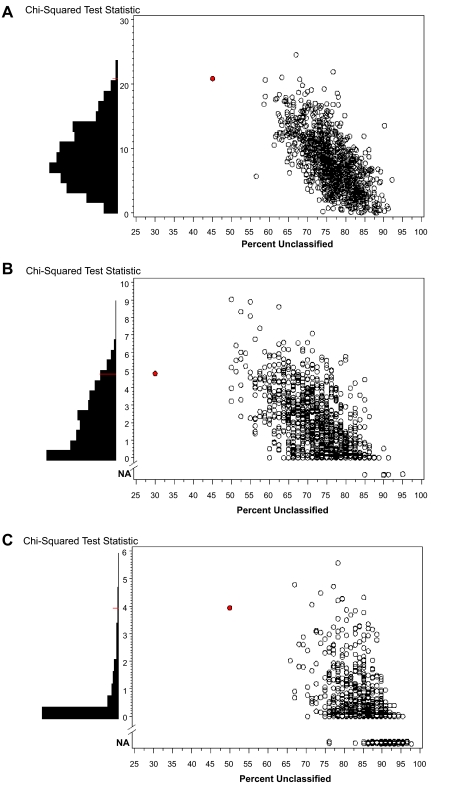
The Cox regression test statistic plotted against the percentage of unclassified samples for each of the 1000 permutations. The circle filled in red denotes the original classification. A test statistic listed as ‘NA’ indicates that the samples were classified into only one class and thus no test statistic could be calculated. The empirical p-value from the chi-squared statistics is depicted as a histogram in the left margin. (A) Glinksy et al. (2004) prostate tumor samples. P < 0.0001; (B) van’t Veer et al. (2002) breast tumor samples, p = 0.0783; (C) Beer et al. (2002) lung tumor samples, p = 0.0111.

Accepting the above significant separation of the Kaplan-Meier curves as validation of the CSR signature in vivo, we proceed to test the classification null hypothesis using 1000 random samples from the genes in common between the in vivo and in vitro samples, see Table 1. The size of the randomly drawn set of genes was determined by the correlation in the original CSR genes, such that the randomly drawn sets contained an equivalent number of effectively independent genes as the CSR set. The TSI score was recalculated on each of these 1000 random gene sets. It was then used to classify the in vivo samples and predict survival. Figure 5 depicts the classification and prediction ability of the 1000 random sets for each of the three in vivo data sets. The CSR gene predictor is colored red in these plots. The vertical axis plots the predictive ability of the gene set as the chi-squared test statistic associated with univariate Cox regression on the classifier. If we look at the vertical margin we arrive at the permutation p-value as depicted by the marginal histogram. However, we have additional information about the utility of the CSR signature as a classifier. The horizontal axis provides the percentage of the samples that remained unclassified in each of the 1000 random sets. In each case, the classifier based on the CSR genes has a lower percentage of unclassified samples than any of the randomly drawn gene sets. Finally, note that for some of the randomly drawn gene sets, see Figures 5B and 5C, the samples were classified into only one group and thus the chi-squared test statistic could not be calculated. This occurred when the percentage of unclassified samples was high.

## Discussion

### TSI based classification shows heterogeneity among samples

As depicted in Figure 2, the simple dichotomization of in vivo samples by hierarchical clustering is far from optimal. By the nature of hierarchical clustering, dichotomization can be achieved by splitting samples at the first node. In Figure 2 we have color coded the samples by their TSI predicted classification (red = serum activated, blue = serum independent, tan = unclassified) and we see that there is heterogeneity in the classification suggested by dichotomization at the first node of the dendrogram. This heterogeneity is apparent in the Kaplan Meier plots of Figure 4. Notice that the prostate samples appear to be least heterogeneous, see Figure 2B, in that most of the serum activated samples are clustered on the left and most of the serum independent samples are clustered on the right with the unclassified samples interspersed among both branches. The Kaplan Meier plot in Figure 4A suggests that those samples which can be classified by their serum response have the best and worst recurrence free survival with the unclassified samples having intermediate recurrence free survival. The intermediate nature of the unclassified samples may be due to a third class of tumors with moderate serum response or it may be due to a blending of high risk and low risk samples that were not separated by the CSR signature.

The breast cancer samples appear to have a more well defined subset of unclassified samples, see Figure 2c. The far right branch of the dendrogram (as split on the second node) contains a high percentage of unclassified samples. In the Figure 4b, the unclassified samples are associated with a recurrence free survival curve that is worse than for the serum activated samples. In the lung cancer data it is not clear that classification on any of the first three nodes of the dendrogram would result in homogeneous classification based on the CSR signature; see Figure 2d. However, using the TSI classification we are able to significantly split the samples into good and bad prognosis groups based on overall survival; see Figure 4c.

### Differences in array configurations may reduce utility of the in vitro signature

One problem encountered in this analysis was the integration of gene expression data across microarray platforms. We attempted to compensate for this numerically by global standardization that centered the array-wise median values at zero. Furthermore, in the TSI algorithm genes were standardized to zero mean and unit standard deviation before being mapped into the reduced space. An additional complication, beyond numerical scaling, is that the differing array configurations between the in vitro and in vivo experiments mean that only those genes with Unigene ID numbers common to both data sets can be considered. This initially excludes ESTs from the in vitro signature as well as other features that do not have Unigene ID numbers. The signature is further reduced by focusing on only the common genes between data sets as determined by Unigene ID. We expect that there is correlation between the genes within the CSR signature and thus the loss of some genes from this signature will be tolerable.

The most dramatic decrease in CSR genes available for the analysis was for the Beer et al. (2002) lung samples which measured only 32.6% of the 484 Unigene mapped CSR genes; see Table 1. It is possible that the high observed percentage of unclassified samples, 51.2%, is related to this diminished in vitro signature. Also, notice that in Figure 3c, that the mapping of the in vitro samples into the reduced space appears to have flipped about horizontal axis from what we saw for the other two in vivo data sets. Since the reduced space is determined by the in vitro data we expect that this inversion is a result of the diminished in vitro signature. However, this inversion does not affect the association of the classification with prognosis. As shown in Figure 4c the serum induced class has worse overall survival than the serum independent class, as expected. This change in the reduced space mapping highlights the necessity to calculate the TSI classifier independently for each in vivo dataset, or particularly for each different array platform and configuration used by the in vivo experiments.

### Permutation plots provide a complete view of the null distribution

Finally, we turn our attention to the permutation testing depicted in Figure 5. These three plots carry a lot of interesting information regarding the utility of the CSR gene signature as a predictor of survival among the three tumor types. First, consider the horizontal axes of Figures 5a–c. It is intriguing that for all three tumor types the CSR signature has the lowest percentage of unclassified samples. Yet we see that percentage of classified samples is not the sole predictor of significant separation in the survival curves since there are randomly selected gene sets that have higher percentages of unclassified samples but also have higher test statistics.

Next, consider the empirical p-value for testing H_0_ ^class^. In the prostate samples, Figure 5a, the empirical p-value is 0.0040, whereas the p-value obtained from a simple training/testing strategy is very small (chi-squared test statistic = 20.89, p < 0.0001). In fact from the scale on the vertical axis we see that most of the random permutation samples were able to predict a significant separation in the survival of the prostate cancer patients. Thus had we relied only on the training/testing strategy we could not distinguish that the CSR signature is superior to 99.6% of the randomly selected signatures. The range of scale of the test statistics for the breast cancer and lung cancer samples are less dramatic. In fact the empirical p-value for the lung cancer dataset behaves we would normally expect, showing that a minimally significant test statistic in the training/testing setting (p = 0.0352) is indeed superior to test statistics generated under the classification null hypothesis (empirical p = 0.0111).

## Conclusions

### Further thoughts on hypothesis testing

Here we have discussed the nature of hypothesis testing when integrating gene expression signatures derived from hypothesis driven in vitro studies with gene expression profiles of in vivo tumor samples. Assigning significance to classification results and associations is necessary to evaluate the utility of the in vitro signature in cancer development and progression as found in vivo. However, for accurate assessment of significance it is necessary to consider the underlying null hypothesis that is being tested. Though the permutation test has been widely accepted as a panacea for significance testing we discussed how the permutation must be done with care so that the underlying null distribution is appropriately reconstructed. We provide a method of assessing the classification potential of an in vitro signature using the TSI classifier of Sandberg and Ernberg (2005). The classification null hypothesis underlies tests of this classifier and thus permutation sampling is available for construction of the null distribution of test statistics.

Although we have discussed the value of a well defined null hypothesis, the interpretation of the alternative hypothesis comes into play when the null is rejected. In particular, the alternative hypothesis for the classification null hypothesis is that at least one predictive signature exists. The number of such signatures is not known and thus the signature being tested need not be unique. We know from Fan et al. (2006) that there are likely to be several predictive signatures in any gene expression study. These may be biologically similar but need not share a substantial number of genes. Rejection of the classification null hypothesis only provides evidence for the existence of one or more predictive signatures. In fact a significant empirical p-value suggests only superiority of the in vitro gene list above the randomly generated gene lists of comparable size. If other in vitro hypotheses were tested the gene signatures generated may also be predictive. Yet, we argue that regardless of its uniqueness, any gene signature that rejects the classification null hypothesis is worthy of further study of its biological relevance.

### Further thoughts on thresholding

We have remarked at several points about the use of thresholding in the algorithms. Arbitrary thresholds used to select genes that are interesting biologically or signatures that are significant statistically may not always be satisfying. We briefly discussed how the concept of GSEA could be adapted to a regression model that would not require a strict definition of the in vitro gene set of interest. Yet, the TSI-type algorithm that we proposed still used thresholding of the in vitro data to produce a signature. This is not necessary for the sake of the algorithm and classification should still be possible using the entire gene signature of the in vitro samples. Ultimately the classifier is built on the correlation of the in vivo samples to composite signatures for each experimental condition in the reduced space.

We again used thresholding in the classification of the in vivo samples by requiring a significant correlation with one of the experimental centroids. The threshold for significance was left at the typical level of p < 0.05, although this could be adjusted to achieve desired specificity and sensitivity in the classifier by dividing the in vivo samples into training and test sets and examining various thresholds. Alternatively, the correlation score could be used as a continuous variable in the Cox regression models. In this way those samples that were not classified in the dichotomous classification would contribute to the model.

## Figures and Tables

**Table 1.** Genes were matched between platforms using Unigene ID numbers. For the permutation testing, all common genes between the in vitro experiment (Chang et al. 2004) and each in vivo experiment were considered (prostate: Glinsky et al. 2004; breast: van’t Veer et al. 2002; lung: Beer et al. 2002). The classification of a set of in vivo samples was done based on only the CSR genes identified in that data set. Permutation sample size was determined based on the effective number of independent genes in the CSR signature.

	Unigene maped genes per microarray	Genes common with in vitro samples	Unigene mapped CSR genes	Effective number of independent genes
In Vitro Samples	20414	—	484	—
Prostate Cancer Samples	11772	9753	367	345
Breast Cancer Samples	17168	13600	421	399
Lung Cancer Samples	4705	3891	158	136

**Table 2.** Cox regression was run on the samples that were classified as serum induced or serum independent by the CSR gene signature. Unclassified samples are excluded from this analysis. The hazard ratios are relative to the serum independent classification.

	**Number Classified**						
	**Serum Induced**	**Serum Independent**	**Percent Unclassified**	**Hazard Ratio**	χ**^2^Test Statistic**	**p-value**	**Empirical p-value**
Prostate							
Cancer	78	83	45.4%	3.35	20.9	<0.0001	0.0040
Samples							
Breast							
Cancer	27	27	30.8%	2.96	4.80	0.0284	0.0783
Samples							
Lung							
Cancer	18	24	51.2%	3.40	3.94	0.0471	0.0111
Samples							
